# Post-truth epistemic beliefs rooted in the Dark Factor of Personality are associated with higher COVID-19 vaccination refusal

**DOI:** 10.1038/s41598-023-31079-9

**Published:** 2023-03-14

**Authors:** Jan Philipp Rudloff, Fabian Hutmacher, Markus Appel

**Affiliations:** Psychology of Communication and New Media, Human-Computer-Media Institute, University of Würzburg, Oswald-Külpe-Weg 82, 97074 Würzburg, Germany

**Keywords:** Psychology, Diseases

## Abstract

A substantial number of people refused to get vaccinated against COVID-19, which prompts the question as to why. We focus on the role of individual worldviews about the nature and generation of knowledge (epistemic beliefs). We propose a model that includes epistemic beliefs, their relationship to the Dark Factor of Personality (D), and their mutual effect on the probability of having been vaccinated against COVID-19. Based on a US nationally representative sample (*N* = 1268), we show that stronger endorsement of post-truth epistemic beliefs was associated with a lower probability of having been vaccinated against COVID-19. D was also linked to a lower probability of having been vaccinated against COVID-19, which can be explained by post-truth epistemic beliefs. Our results indicate that the more individuals deliberately refrain from adhering to the better argument, the less likely they are vaccinated. More generally, post-truth epistemic beliefs pose a challenge for rational communication.

## Introduction

For thousands of years, humans have tried to define and understand what constitutes truth and knowledge^1^. In the contemporary social sciences, there is a wide consensus that even though knowledge is always rooted in historical and societal circumstances, some allegations or opinions are more valid than others because they are backed by better evidence, for example by scientific data^2–4^. In contrast, there have been intellectual movements since antiquity assuming that there is no such thing as truth at all or at least claiming that it is not the better but the rhetorically more appealing argument that will prevail in debates. These movements, which have commonly been portrayed as relativistic or sophistic in nature^5^, seem to hold that what counts as truth is ultimately a matter of power. Consequently, they often share unbounded constructivist perspectives that challenge the very idea of any scientific consensus^3,6^. Individuals who doubt the possibility of truth and knowledge will ignore evidence, believe what feels true to them and will not be open to arguments challenging their worldview^7,8^. As we argue, these worldviews are crucial to the interpretation of information and ultimately to individual behavior such as getting vaccinated.

### Epistemic beliefs

In psychological research, worldviews about the concept of knowledge are referred to as *epistemic beliefs*^9^. They are developed during childhood and adolescence through socialization and are considered to be relatively stable albeit not fixed over time^7,10^. Typically, they are assumed to develop from so-called *naïve* to *sophisticated* beliefs^11^. Kuhn et al.^12^ distinguish between three different stages of epistemic beliefs. In the beginning, individuals hold an absolutist perspective, which implies that knowledge is an objective entity being located in the outside world that can be perceived with absolute certainty. Later, individuals acknowledge that others may perceive the same event, object, construct or statement differently and therefore develop different opinions (multiplist perspective). The third and last stage is called the evaluativist level and reintegrates both the objective and subjective elements of knowing. In this stage, individuals share a sophisticated and complex understanding of truth. There is still no final consensus on the exact relation between epistemic beliefs and metacognitions: Some authors argue that epistemic beliefs are metacognitions^13,14^, whereas others see them as closely related to but still conceptually distinct from metacognitions^11^. Either way, epistemic beliefs accelerate or inhibit a rational processing of information and are thus related to the accuracy of individuals’ opinions^7,15^.

Our work is based on the framework by Garrett and Weeks^7^ that distinguishes three aspects of epistemic beliefs. First, *Faith in Intuition for Facts* captures how much people rely on their gut feeling when evaluating the accuracy of information. Intuition can be a valuable resource in decision-making^16,17^, especially when being followed by analytic thinking. Prioritizing one’s intuition, however, bears the risk of ignoring and disregarding existing evidence, which may lead to drastic misperceptions^18^. Second, *Need for Evidence* refers to the degree to which people find it necessary to align their opinions with the known facts, for example externally validated data. People who put little emphasis on evidence are prone to making decisions based on ideological convictions, regardless of the current scientific consensus^19,20^. Third, *Truth is Political* refers to the degree to which people are convinced that facts are dependent on the societal and political context, that is, that facts are shaped by those in power, for example, politicians, journalists, and scientists. In extreme cases, it may be concluded that what counts as “truth” is nothing but a matter of power^2,7^.

A strong Faith in one’s Intuition for Facts, a low Need for Evidence and the strong conviction that Truth is Political, have been referred to as *post-truth epistemic beliefs*^8^*.* This distinct combination of beliefs prevents people from questioning their opinions and immunizes them against any external intervention. Individuals with pronounced post-truth epistemic beliefs will deliberately choose to disregard evidence and instead believe what they intuitively hold to be true. Note that the notion of post-truth epistemic beliefs does not imply a dichotomy of sophisticated epistemic beliefs on the one hand and problematic post-truth epistemic beliefs on the other^1,21–25^. Instead, epistemic beliefs are understood to be varying along a continuum^7^. Post-truth epistemic beliefs have been associated with lower education and lower need for cognition, which is one’s desire for cognitively demanding tasks^7^. They have also been linked to the endorsement of COVID-19 conspiracy theories as well as problems with discerning fake news from real news^8,26^.

### The Dark Factor of Personality

Prior research has shown that post-truth epistemic beliefs are embedded in a broader personality disposition, the *Dark Factor of Personality* (D)^8,26^. It is defined as “the general tendency to maximize one’s individual utility—disregarding, accepting, or malevolently provoking disutility for others—accompanied by beliefs that serve as justifications” (p. 657)^27^. Utility refers to various forms of material success, but also includes rewarding feelings, such as power or pleasure. Moreover, utility does not necessarily refer to *actual* mid- to long-term benefits (e.g. economic prospering or well-being), but rather *perceived* benefits resulting from ethically questionable behavior (e.g. feeling superior to others). D is considered to be the core of all dark traits, for example egoism, Machiavellianism, and psychopathy and explains their common variance^27^. Individuals high in D show a variety of ethically questionable behavior, such as lying or exploiting and manipulating others^27^. They manage to uphold a positive self-image despite their malevolent behavior by relying on beliefs and rationalizations that help to justify it^28^. Crucially, “the concept of D does not imply that individuals must hold any one particular belief or set of beliefs; instead, the main idea is that individuals hold some belief(s) that they deem appropriate to justify malevolent acts” (p. 4)^29^. Thus, they may embrace a relativistic and cynical worldview, enabling them to bend morals and norms, whenever they perceive it to be beneficial^27,30,31^. Importantly, prior research has shown that individuals with high levels in D tend to endorse post-truth epistemic beliefs^8,26^. These beliefs are deemed advantageous because they justify not adhering “to the unforced force of the better argument” (p. 305)^32^. If, as previously shown, D was associated with post-truth epistemic beliefs, D should in turn be associated with lower adherence to recommendations that are based on rational communication. The higher D, the less likely individuals should be convinced by expert sources and strong arguments. In the case of the COVID-19 pandemic, this should result in less adherence to behavioral recommendations expressed by health organizations and the government^8^. Moreover, evidence suggests that dark traits are linked to engaging in risky behavior^33^, opposing laws and authorities^29,34^, and ignoring the interests of vulnerable others^35,36^. Post-truth epistemic beliefs likely accompany and fuel basic behavioral tendencies as justifications at different steps of the motivational process culminating in a decision.

### COVID-19 vaccination

Vaccination programs are one of the most efficient public health measures and have contributed to reducing morbidity and mortality rates of a variety of different infectious diseases^37^. To be successful, that is, to reach protection for entire communities, vaccination programs require a high uptake level, which can only be achieved if people trust those who advocate vaccination: health professionals, policymakers, the media, and scientists^38^.

This general pattern also applies to the COVID-19 vaccination in particular. Even though COVID-19 vaccines such as the Moderna or the Pfizer-BioNTech COVID-19 vaccine are considered safe and effective by now^39–41^ and are typically portrayed as such in mass media^42^, some people seem to ignore the available evidence. We argue that post-truth epistemic beliefs prevent individuals from questioning their opinions and immunize them against any external intervention, such as arguments that favor vaccine uptake based on scientific evidence.

With regard to dark traits, studies have shown associations with less health-protective behavior against COVID-19^36,43–45^, including vaccination hesitancy^46–48^. However, these studies on vaccination hesitancy have at least three shortcomings: First, they typically relied on convenience samples. Second, the vast majority of psychological research on vaccine uptake focuses on self-report measures of vaccination *intention* rather than the actual vaccination *status*^37,49^. Third, they did not investigate D as a potential underlying factor driving post-truth epistemic beliefs.

### The current research

We conducted a study to address these shortcomings. In order to do so, we relied on a large US nationally representative sample, which enables us to ensure the robustness of our results and to draw conclusions that can be generalized for the US American public. In many Western countries such as the USA, the primary immunization phase against COVID-19 can be considered complete. That is, at the time of assessment (July 2022), almost anyone willing to get vaccinated against the original variant of the virus has received their vaccination doses^50^. Thus, it is the ideal time to assess individuals’ actual vaccination *status* instead of their vaccination *intention* and to analyze the reasons behind uptake or refusal in order to gain insights for future vaccination campaigns. Moreover, we propose a model that includes the Dark Factor of Personality as an antecedent of post-truth epistemic beliefs and a lower probability of having been vaccinated against COVID-19 as a consequence.

**Figure 1:**
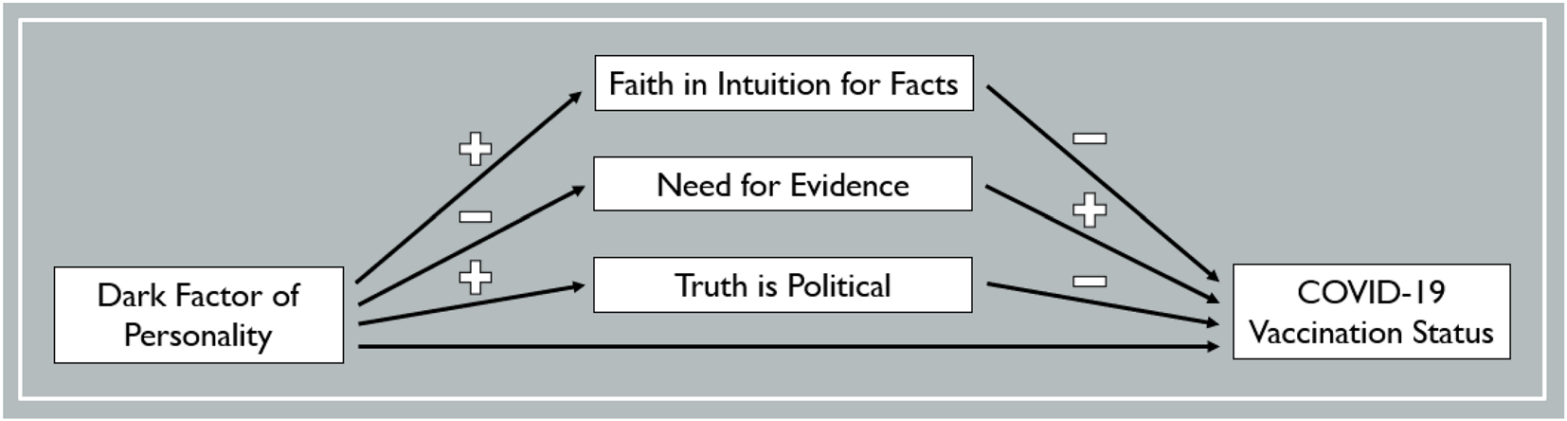
The Proposed Mediator Model with COVID-19 Vaccination Status as the Dependent Variable.

More specifically, we propose three major hypotheses: First, post-truth epistemic beliefs, which comprise a strong Faith in Intuition for Facts, a low Need for Evidence, and the strong conviction that Truth is Political, should predict a lower probability of having been vaccinated against COVID-19. Second, the Dark Factor of Personality should predict holding post-truth epistemic beliefs. Third, these associations should result in a mediation effect as depicted in Fig. 1. We will use two indicators of COVID-19 vaccination status: (1) whether individuals have been vaccinated against COVID-19 at all (i.e. received at least one vaccine dose) and (2) whether individuals have been fully vaccinated (i.e. received at least two vaccine doses). If it turned out that D and post-truth epistemic beliefs indeed drive refusal to get vaccinated, that would be a (worrying) explanation why some individuals act in contrast to the scientific consensus. In the case of at least some individuals, arguments must fail as these individuals are simply not willing to adhere to the better argument.

## Results

In our sample (*N* = 1368), 239 participants (18.8%) had received no COVID-19 vaccination, 75 participants (5.9%) had been vaccinated once, 290 participants (22.9%) had been vaccinated twice, 497 participants (39.2%) had been vaccinated 3 times, and 167 participants (13.2%) had been vaccinated four or more times against COVID-19.

**Figure 2:**
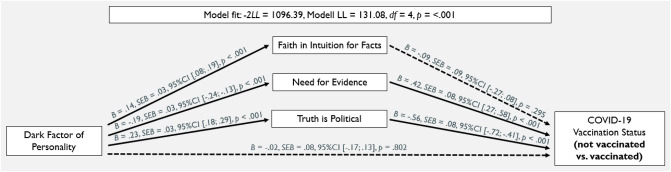
Main Results of the Parallel Mediator Model with COVID-19 Vaccination Status as the Binary Dependent Variable (0 = not vaccinated; 1 = vaccinated). Solid paths indicate significant associations (*p* < 0.05), dashed paths are non-significant.

We conducted two parallel multiple logistic mediation analyses using PROCESS version 3.4.1^51^. For the first analysis, Faith in Intuition for Facts, Need for Evidence and Truth is Political were included as simultaneous mediators. The Dark Factor of Personality served as the predictor variable influencing the binary dependent variable COVID-19 vaccination status (not vaccinated/vaccinated at least once) directly and indirectly through the three mediators^51^. Figure 2 shows all path coefficients, standard errors, and *p*-values. As can be seen in Table 1, all major variables were significantly correlated in the expected directions. D significantly predicted whether (1) or not (0) participants had been vaccinated at least once against COVID-19, *B* = -0.20; *SEB* = 0.07; Wald (1) = 8.84, *p* = 0.003, OR = 0.815. The likelihood-ratio test was significant, *− 2LL* = 1096.39, Model LL = 131.08, *df* = 4, *p* < 0.001 (McFadden’s *R*^*2*^ = 0.11, Cox & Snell’s *R*^*2*^ = 0.10, Nagelkerke’s *R*^*2*^ = 0.16), indicating that our model significantly predicted the participants’ COVID-19 vaccination status. Need for Evidence and Truth is Political were significantly associated with participants’ COVID-19 vaccination status in the expected directions. Faith in Intuition for Facts was no significant predictor. D was significantly associated with all epistemic belief subscales in the expected directions. Thus, we found significant indirect effects for Need for Evidence, *B* = − 0.08, *SEB* = 0.02, 95% CI [− 0.12; − 0.05], and for Truth is Political*, B* = − 0.13, *SEB* = 0.03, 95% CI [− 0.19; − 0.09], whereas Faith in Intuition for Facts was no significant mediator, *B* = − 0.01, *SEB* = 0.01, 95% CI [− 0.04; 0.01].

**Table 1:** Means, Standard Deviations, and Zero-Order Correlations of the Continuous Variables.

	*M* (*SD*)	(1)	(2)	(3)	(4)
(1) Dark factor of personality	2.18 (0.79)	–			
(2) Faith in intuition for facts	4.63 (1.18)	0.14**	–		
(3) Need for evidence	5.75 (1.02)	− 0.19**	− 0.37**	–	
(4) Truth is political	3.31 (1.48)	0.24**	0.23**	− 0.32**	–
(5) COVID-19 vaccination status	–	− 0.08*	− 0.14**	0.25**	− 0.27**

*N* = 1268. ** *p* < 0.001, * *p* < 0.01. The Dark Factor of Personality and the epistemic beliefs subscales were measured on a 7-point scale that ranged from *strongly disagree* (1) to *strongly agree* (7). COVID-19 vaccination status was operationalized as a binary variable (0 = not vaccinated; 1 = vaccinated).

**Figure 3:**
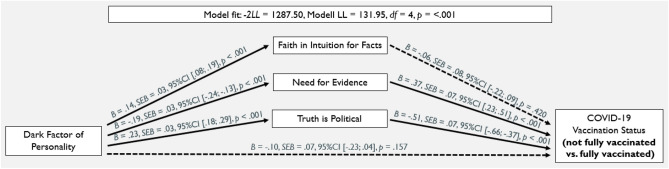
Main Results of the Parallel Mediator Model with COVID-19 Vaccination Status as the Binary Dependent Variable (0 = not vaccinated or partially vaccinated; 1 = fully vaccinated). Solid paths indicate significant associations (*p* < 0.05), dashed paths are non-significant.

In order to test the robustness of our results, we examined whether our model could also predict the probability of being fully vaccinated, that is, having received at least two doses^52^. Thus, we performed a second analysis for having been fully vaccinated (1) and not having been vaccinated or only once (0). As can be seen in Fig. 3, results remained virtually identical to the previous analysis. In sum, the study provides evidence that post-truth epistemic beliefs predict whether participants have been (fully) vaccinated against COVID-19 and that they explain the link between the Dark Factor of Personality and the COVID-19 vaccination status.

## Discussion

The COVID-19 pandemic highlights the need to understand why a substantial number of individuals are unwilling to get vaccinated despite an enormous body of scientific evidence speaking to the effectiveness and safety of the vaccines^39–41^. Our study yields three important results: First, epistemic beliefs predicted whether or not individuals were vaccinated against COVID-19. The stronger participants endorsed post-truth epistemic beliefs, the lower the probability that they had been vaccinated. This finding complements research showing that post-truth epistemic beliefs are linked to COVID-19 conspiracy beliefs^8^. Second, the stronger individuals’ Dark Factor of Personality, the lower the probability that they had been vaccinated. This is in line with prior research on individual dark traits and vaccination *hesitancy*^46–48^.

Third, post-truth epistemic beliefs explain the link between D and individuals’ COVID-19 vaccination status. Importantly, all results were virtually identical for different definitions of having been vaccinated (having received at least one dose versus having received two or more doses). Thus, as expected D was associated with lower adherence to recommendations that are based on rational communication such as expert sources and strong arguments. More precisely, this implies that the decision-making process of individuals high in D is guided by post-truth epistemic beliefs, which help to fend off information advocating vaccine uptake. As a consequence of not adhering to rational arguments, they tend to remain unvaccinated.

With regard to the perceived benefits of vaccine uptake, these individuals should only be willing to get vaccinated if it they perceive it to be advantageous, for example if they belong to a risk group or their employer demands them to get vaccinated. As outlined in the introduction, *perceived* individual utility does not necessarily go hand in hand with *actual* utility. That is, vaccination refusal could have a high *perceived* individual utility as it fuels, for instance, a feeling of superiority (“I am strong enough, I do not need the vaccine”;^53^) or the feeling of being smarter than the majority (“I understand things about this vaccine that others do not”). This does not imply, of course, that the deliberate refusal to adhere to rational arguments in favor of vaccine uptake is not an ill-advised decision that puts the individual’s health at risk. In this context also note that a habitual unwillingness to base one’s decisions on evidence and rational arguments could additionally lead to a reduced ability to do so over time due to a lack of practice^26^. This should also decrease the ability to assess which behavioral option is the most beneficial.

Taken together, our study also has broader implications for understanding the deeper reasons behind problematic information processing and decision-making. Psychological research often focuses on cognitive biases^54–57^ or thinking styles^58,59^ to explain seemingly irrational cognition and behavior such as belief in misinformation, climate change denial or vaccination hesitancy. Our research complements these perspectives by highlighting the insight that getting vaccinated against COVID-19 may not solely be a function of one’s access to the right information, nor of one’s ability to process it rationally, but of one’s willingness to adhere to the better argument. In other words, the stronger people’s post-truth epistemic beliefs the more likely they disregard the quality of arguments.

### Practical implications

A belief system in which the evaluation of arguments against criteria of truth is deliberately suspended presents a major challenge to health communication, and more broadly speaking, to rational communication in other fields. As disillusioning as this realization may be, it also bears the opportunity for two potentially fruitful countermeasures. First, preventing the development of post-truth epistemic beliefs should be of vital interest to our society. Efforts must be increased to emphasize the difference between mere opinions and scientific evidence and the importance and challenges of rational communication. As epistemic beliefs are developed during childhood and adolescence^7,10^, schools could be the appropriate institutions to implement these measures. Second, the results underscore the relevance of providing incentive structures for rational behavior, in a sense that behavior that is in line with scientific evidence should also be the behavioral option with the highest perceived personal benefits^60,61^.

### Limitations and open questions

There are certain limitations to our study. First, note that despite the significant zero-order correlation between Faith in Intuition for Facts and participants’ COVID-19 vaccination status (Table 1), the link was not significant in the mediation model. From a theoretical perspective, the effect of Faith in Intuition for Facts on vaccine uptake might depend on the type of media individuals consumed and the intuitions formed during the pandemic. Individuals who mainly followed mainstream media were most likely most exposed to the view that the vaccines are safe and effective. Hence, they may have developed an intuition that the vaccines can be trusted. Conversely, individuals who mainly followed alternative media were likely primarily exposed to the view that vaccines cannot be trusted and might have adjusted their intuitions accordingly. Therefore, Faith in Intuition for Facts should have no consistent association with COVID-19 vaccination status. Additionally, there is also a potential statistical explanation for the non-significant link in the mediation model: Faith in Intuition for Facts showed the smallest zero-order correlation with COVID-19 vaccination status of all epistemic beliefs subscales. Thus, the non-significant path in the mediation model could be due to the common variance shared by D, Faith in Intuition for Facts, Need for Evidence, and Truth is Political as suggested by the significant associations between all three mediators as well as D (Table 1). Second, our design is cross-sectional, so we cannot conclude causality neither for the link between D and epistemic beliefs, nor for their associations with individuals’ COVID-19 vaccination status. However, we would like to stress that it is much more likely that personality traits such as the Dark Factor of Personality influence epistemic beliefs than the other way round. The same applies to the links between D, post-truth epistemic beliefs, and individuals’ COVID-19 vaccination status. We encourage future research to address the issue of causality. Third, the study focuses on post-truth epistemic beliefs as a predictor of participants’ COVID-19 vaccination status. Our approach does not exclude, but rather complements alternative perspectives, for example work on a lack of scientific trust^62,63^ or conspiracy beliefs^64^. Further, several studies have indicated that dark traits directly influence COVID-19 related behaviors, such as vaccination hesitancy, or non-compliance with measures and recommendations. These findings are often interpreted as a result of low caring for others^36^ or increased risk-taking in health-related behaviors in general^47^. Our study does not contradict these findings, but rather provides an additional explanation for seemingly irrational cognition and behavior, which could result from poor judgment due to deliberately disregarding evidence and rational arguments as post-truth epistemic beliefs accompany and guide decision-making^26^. Third, individuals high in D are prone to deceptive behavior, so we cannot rule out that they may have purposefully misreported their COVID-19 vaccination status. However, such a tendency should induce unsystematic error rather than driving the observed effects.

### Conclusion

In sum, our study highlights the pivotal role of post-truth epistemic beliefs in explaining why a substantial number of people remained unvaccinated against COVID-19. People with post-truth epistemic beliefs seem to be unwilling to adhere to the overwhelming scientific evidence in favor of vaccine uptake. This finding provides the ground for potential measures to increase peoples’ willingness to act in accordance with the scientific consensus. The fact that epistemic beliefs are developed during childhood and adolescence presents a window of opportunity for interventions, yet at the same time emphasizes the need to act foresightedly.

## Method

In reporting our study, we follow the Journal Article Reporting Standards^65^. Sample size determination, all data exclusions as well as all measures used in the study are reported in the following. Data, analysis code and research materials are available at 10.17605/OSF.IO/Z8UQ5. The study was preregistered (https://aspredicted.org/359tb.pdf) and has been approved by the internal review board of our institution. Informed consent was obtained from all participants.

### Ethics approval statement

The study followed the ethical guidelines of the APA and the German Psychological Society (DGPs) and was approved by the internal review board of the Human–Computer-Media Institute at the University of Würzburg.

### Participants

We recruited a US nationally representative sample via Prolific, which means that distributions of gender, age, and ethnicity correspond to the US census. As we expected that a substantial number of participants would not disclose their vaccination status, we aimed for 1300 participants to account for potential exclusions. In total, 1304 participants completed the questionnaire from July, 26–29, 2022, and were paid 0.92$, which corresponds to an average hourly wage of 9.32$. The following exclusion criteria were applied: First, nine participants were excluded because they did not respond correctly to a control item (e.g. “This is a control question. Please select ‘strongly disagree’”). As preregistered, we checked for extremely low response times, but all participants exceeded the defined threshold of 120 s to complete the study. This threshold was based on the idea that people need at least 2–3 s to answer survey items diligently^66^. Participants were also asked to provide a brief description of the study in English to check whether they were native speakers and responded diligently. As a consequence, four participants were excluded. Another 10 participants were excluded because they were under the age of 18 or did not indicate their age. Finally, we excluded 13 participants, because they did not disclose their COVID-19 vaccination status. Our final sample consisted of 1268 participants (*M* = 45.51 years, *SD* = 16.30 years, 18–93 years, 50.2% female, 48.5% male, 1.3% unspecified or another gender identity). With regard to educational attainment, 30.2% had a high school diploma, 45.0% had a bachelor’s degree, 17.3% had a master’s degree, and 3.2% a Ph.D., while 4.3% completed some high school or trade school. Regarding ethnicity, 78.1% were White (including Hispanic/Latinx Americans), 12.5% were Black, 5.6% were Asian, 1.8% were Mixed and 2.1% indicated “Other” or did not disclose their ethnicity.

### Measures

#### Epistemic beliefs

We relied on a 12-item questionnaire developed by Garret and Weeks^7^ to measure participants’ epistemic beliefs. It captures three subscales comprising four items each: Faith in Intuition for Facts (e.g. “I trust my gut to tell what’s true and what’s not”, α = 0.90), Need for Evidence (e.g. “Evidence is more important than whether something feels true”, α = 0.87), and Truth is Political (e.g. “Facts depend on their political context”, α = 0.88). Items were answered on a 7-point scale that ranged from *strongly disagree* (1) to *strongly agree* (7).

#### The dark factor of personality

We measured the Dark Factor of Personality using the D16 short version by Moshagen et al.^29^. It contains 16 items that were answered on a 7-point scale that ranged from *strongly disagree* (1) to *strongly agree* (7) (e.g. “My own pleasure is all that matters”, α = 0.89).

#### COVID-19 vaccination status

Further, we assessed participants’ COVID-19 vaccination status (“Please indicate how many times you have been vaccinated against COVID-19”). Options were “0”, “1”, “2”, “3”, “4 or more”, and “I don’t want to answer”. For the first analysis, we aggregated all options other than “0”, thereby differentiating solely between individuals who were willing to get vaccinated (scored 1 in the analyses) from those who were not (scored 0). For the second analysis, we aggregated the options “2”, “3” and “4 or more” to indicate full vaccination status (score 1), whereas “0” and “1” indicated no full vaccination status (score 0). We also asked participants to indicate which vaccine they had received (“Moderna COVID-19 vaccine”, “Pfizer-BioNTech COVID-19 vaccine”, “Janssen (Johnson and Johnson) COVID-19 vaccine”, “Novavax COVID-19 vaccine”, “Other”, “I can’t remember”). Selecting multiple options was possible. The items concerning individuals’ COVID-19 vaccination status were embedded in several additional items about participants’ travel activities in the past 2 years to disguise the purpose of the study and to avoid reactance. Participants were thoroughly debriefed at the end of the study.

#### Political affiliation

Participants were asked to indicate whether they self-identified as Republicans (*n* = 277), Democrats (*n* = 582) or Independents (*n* = 408). One participant did not indicate their political affiliation.

## Data Availability

The study underlying the manuscript was preregistered (https://aspredicted.org/359tb.pdf), the manuscript reports original data and is not under review elsewhere. The data, codes, and the online supplement can be found at 10.17605/OSF.IO/Z8UQ5. These links are also provided in the methods section. APA ethical standards were followed in the conduct of the reported studies and the study was approved by the internal review board of the Human–Computer-Media Institute, University of Würzburg, Germany.
